# Characteristics and Outcome for Persons with Diabetic Foot Ulcer and No-Option Critical Limb Ischemia

**DOI:** 10.3390/jcm9113745

**Published:** 2020-11-21

**Authors:** Marco Meloni, Valentina Izzo, Valerio Da Ros, Daniele Morosetti, Matteo Stefanini, Enrico Brocco, Laura Giurato, Roberto Gandini, Luigi Uccioli

**Affiliations:** 1 Department of Systems Medicine, University of Tor Vergata, Viale Oxford 81, 00133 Rome, Italy; valentina_izzo@virgilio.it (V.I.); lauragiurato@yahoo.it (L.G.); luccioli@yahoo.com (L.U.); 2 Department of Biomedicine and Prevention, University of Tor Vergata, Viale Oxford 81, 00133 Rome, Italy; darosvalerio@gmail.com (V.D.R.); danielemorosetti@hotmail.com (D.M.); 3 Department of Radiology, Policlinico Casilino, Via Casilina 1049, 00169 Rome, Italy; matteostefanini@hotmail.com; 4 Department of Foot and Ankle, Polyclinic Abano Terme, 35031 Abano Terme, Italy; enbrocco@gmail.com; 5 Department of Interventional Radiology, University of Tor Vergata, Viale Oxford 81, 00133 Rome, Italy; roberto.gandini@fastwebnet.it

**Keywords:** diabetic foot, foot ulceration, limb ischemia, lower-limb amputation, revascularization

## Abstract

The study aimed to evaluate clinical and vascular characteristics, as well as outcomes, for diabetic persons with foot ulceration and no-option critical limb ischemia (CLI). The study group included a sample of patients admitted to our diabetic foot unit because of a new diabetic foot ulcer and CLI. All subjects were managed using a limb salvage protocol which includes lower-limb revascularization. According to whether or not the revascularization procedure was a success, patients were respectively divided into two groups: successfully treated CLI patients (ST-CLI) and no-option CLI patients (NO-CLI). Failed revascularization was considered in the case of technical recanalization failure of occluded vessels (inability to overcome the obstruction) and/or absence of arterial flow to the foot. Limb salvage, major amputation, and death after 1 year of follow-up were evaluated and compared between the two groups. Overall, 239 patients were included, 74.9% belonging to ST-CLI and 25.1% to NO-CLI. NO-CLI patients reported more cases of ischemic heart disease (80 vs. 62.1, *p* = 0.008), heart failure (63.3 vs. 32.4%, *p* < 0.0001), and end-stage renal disease (ESRD) (60 vs. 25.7%) than ST-CLI patients. In addition, more vessels were affected in the NO-CLI group (5.2 ± 1.6 vs. 4 ± 1.5, *p* < 0.0001), and there was more involvement of tibio-peroneal trunk (50 vs. 30.2%, *p* = 0.006), anterior tibial (93.3 vs. 82.7, *p* = 0.03), posterior tibial (93.3 vs. 73.7%, *p* = 0.0005), peroneal (70 vs. 48%, *p* = 0.002), and below-the-ankle arteries (73.3 vs. 39.1%, *p* < 0.0001) than ST-CLI. The 1 year outcomes for the whole population were 69.9% limb salvage, 10.9% major amputation, and 19.2% death. The outcomes for NO-CLI and ST-CLI were, respectively, as follows: limb salvage (13.8 vs. 73.4%, *p* < 0.0001), amputation (30 vs. 4.5%, *p* = 0.0001), and mortality (50 vs. 8.9%, *p* < 0.0001). NO-CLI patients showed a more severe pattern of peripheral arterial disease (PAD) with distal arterial lesions and worse outcomes than ST-CLI.

## 1. Introduction

Currently, a specific limb salvage protocol including lower-limb revascularization for managing ischemic diabetic foot ulcers (DFUs) is well established [1]. 

In recent years, several studies appeared to demonstrate improved rates of limb salvage associated with revascularization in comparison to the medical treatment in patients with peripheral arterial disease (PAD) and DFUs [2]. 

Nonetheless, in clinical practice, several cases of technical revascularization failure, defined as the inability to overcome vessel obstruction and/or absence of blood flow to the foot, are still reported [3,4]. Patients with untreatable critical limb ischemia (CLI), termed no-option critical limb ischemia (NO-CLI), are currently a clinical challenge for all clinicians involved in the management of ischemic DFUs, due to the fact that failed or missing revascularization is a predictor of nonhealing, amputation, and mortality [5]. Nonetheless, even though the needs of patients with NO-CLI remain unmet clinically, there is limited data about specific vascular and clinical features. Therefore, it would be very useful to know the characteristics of NO-CLI patients, in order to evaluate future therapeutic options and their outcomes.

Accordingly, this study aims to evaluate the characteristics and outcomes for patients with ischemic DFUs and NO-CLI, in comparison to patients who have had successful revascularization.

## 2. Materials and Methods

This study is a retrospective cohort study. The sample was composed of conveniently acquired patients who were admitted to our diabetic foot unit because of a new DFU and CLI requiring hospitalization from January 2016 to December 2018.

Data were collected in a local database and retrospectively analyzed. At admission, patients provided consent for the recording and use of their clinical data.

The study was ethically approved and done in accordance with a local ethics committee policy. 

All subjects included were managed by a limb salvage protocol, which includes surgical debridement, antibiotic therapy in the case of infection, offloading of the affected foot, revascularization, and management of their general condition in accordance with international guidance [1].

After hospitalization, patients were regularly followed up in our diabetic foot clinic until wound healing or a different outcome was achieved. Baseline demographic, clinical, ulcer, and vascular findings were recorded. 

Patients who were not suitable or eligible to be treated with lower-limb revascularization, due to critical general conditions or unsalvageable foot at admission, were excluded.

### 2.1. Medical Findings

Ischemic heart disease (IHD) was considered in the case of previous acute coronary syndrome or coronary revascularization, evidence of angina, significant changes in the electrocardiography (above- or under-leveling ST, q wave, inversion of T wave, new left bundle branch block). Cerebrovascular disease was considered in the case of previous cerebrovascular ischemia, previous carotid revascularization, or significant carotid artery disease (occlusion >70%). Hypertension was considered in the case of persistent high blood pressure >130/80 mmHg or current antihypertensive therapy; hypercholesterolemia was defined as low-density lipoprotein (LDL) cholesterol >70 mg/dL or needing statin therapy; patients were only considered smokers if they had a smoking habit at the time of treatment [6]. Anemia was defined as Hb values <13 gr/dL for men and < 12 gr/dL for women. Dialysis was considered in the case of end-stage renal disease requiring renal replacement therapy.

### 2.2. Ulcer Characteristics

Ulcer characteristics are those which were reported during the first assessment at our diabetic foot unit. Ulcer duration was reported in weeks. Deep ulcers were considered in the case of full-thickness skin lesions, extending from the subcutis to tendon, muscle, or bone. Diagnosis of infection was carried out according to clinical signs (redness, warmth, swelling, induration, tenderness, pain, and purulent secretion) and treated, firstly with broad-spectrum antibiotic therapy and then therapy based on culture results if required [1].

All patients received therapeutic shoes for relieving pressure and trauma in the ulcer area during the acute phase. Off-loading was adapted according to both ulcer location and individual needs [1].

### 2.3. Vascular Findings

Diagnosis of CLI was performed according to clinical signs (ulceration or gangrene) and transcutaneous oxygen pressure (TcPO_2_) (<30 mmhg) [7]. Patients with CLI and foot lesions underwent lower-limb revascularization to allow restoration of foot perfusion. Before revascularization, ultrasound duplex was performed to investigate the localization of peripheral arterial lesions; alternatively, if required, computer tomography or magnetic resonance imaging were performed. The revascularization procedure was decided upon with respect to the foot condition, vessels affected, and the patient’s general condition; the endovascular technique was performed for elderly and frail patients with several comorbidities, reduced life expectancy, and significant foot tissue involvement, while open revascularization was indicated in the case of the involvement of the common femoral artery and its bifurcation, or extremely long occlusions (as per mutual agreement among diabetologists, interventional radiologists, and vascular surgeons) of the femoral–popliteal and infra-popliteal arteries [8]. The main aim of revascularization was to open all occluded arteries or, if not technically possible, the revascularization of a targeted artery (wound related artery revascularization) [8]. 

Technical failure of the revascularization procedure was considered in the case of technical recanalization failure of stenosis or occluded vessels (defined as the inability to overcome obstruction) and/or absence of arterial flow to the foot, despite the revascularization intervention. Patients who had failed revascularization were considered no-option CLI patients. Patients were treated with dual antiplatelet therapy (acetylsalicylic acid 100 mg and clopidogrel 75 mg once a day) before the procedure and for at least 1 month after [8]. When aspirin or clopidogrel were not tolerated, ticlopidine was administered. 

Localization of arterial lesions evaluated through retrospective analysis of individual angiograms was described. The monitoring of peripheral blood perfusion by transcutaneous oxygen pressure (TcPO_2_) at baseline and 1 month after revascularization was reported.

According to the technical success or failure of the revascularization procedure, patients were respectively divided into two groups: successfully treated CLI patients (ST-CLI) and NO-CLI patients. 

### 2.4. Outcomes

Limb salvage, major amputation, and death after 1 year of follow-up were evaluated. The first outcome achieved was the only outcome considered. 

Limb salvage was considered in the case of healed patients or nonhealed patients not requiring major amputation during the follow-up; major amputation was considered in the case of any amputation above the ankle.

Demographic, clinical, vascular characteristics, and outcomes were compared between ST-CLI and NO-CLI. 

### 2.5. Statistical Analysis

Statistical analysis was performed using SAS (JMP12; SAS Institute, Cary, NC, USA) on a personal computer. The main reference variable (or main outcome of interest) was “major amputation of the lower limb”; thus, the relative sample size of 239 patients was subject to the following probabilistic considerations: rejection of the null hypothesis was proposed if the average of the population (the average number of amputations), in which the random sample size was taken, differed in absolute value by a quantity equal to or greater than 39% of the standard deviation. To verify this hypothesis, Student’s *t*-test was used with an alpha code = 0.05 (type I error) and beta code = 0.10 (type II error), resulting in 90% test power. Given these probabilistic considerations, the minimum overall number of patients enrolled in the study was 239 subjects.

Data were expressed as the mean ± SD. Comparisons between groups were reported using a Χ^2^ test (frequency data) or Student’s *t*-test (continuous data). A *p*-value < 0.05 as considered statistically significant.

Univariable logistic regression analyses were performed for all potential predictor variables with the outcome of interest (major amputation and mortality), with values presented as univariable odds ratios (ORs) along with the respective 95% confidence interval (CI). Second, all potential predictors were entered simultaneously into a multivariable logistic regression model. These models yielded a set of variables that best predict outcome.

## 3. Results

The study group was composed of 239 patients: 179 (74.9%) belonging to the ST-CLI cohort and 60 (25.1%) to the NO-CLI cohort.

Baseline demographic, clinical, and ulcer characteristics of the whole population, ST-CLI patients, and NO-CLI patients are reported in Table 1. NO-CLI patients reported more cases of ischemic heart disease, heart failure, and end-stage renal disease (ESRD) than ST-CLI patients. 

Distribution of arterial lesions and vascular findings are reported in Table 2. The NO-CLI group showed more involvement of below-the-knee (BTK) (anterior and posterior tibial artery, peroneal artery) and below-the ankle (BTA) arteries (pedal and plantar arteries).

Six patients (2.5%) were treated with surgical revascularization, while 234 (97.5%) were treated with the endovascular technique. Forty-one patients (16.7%) had surgically failed revascularization, while 59 (25.2%) had endovascular failed revascularization.

**Figure 1:**
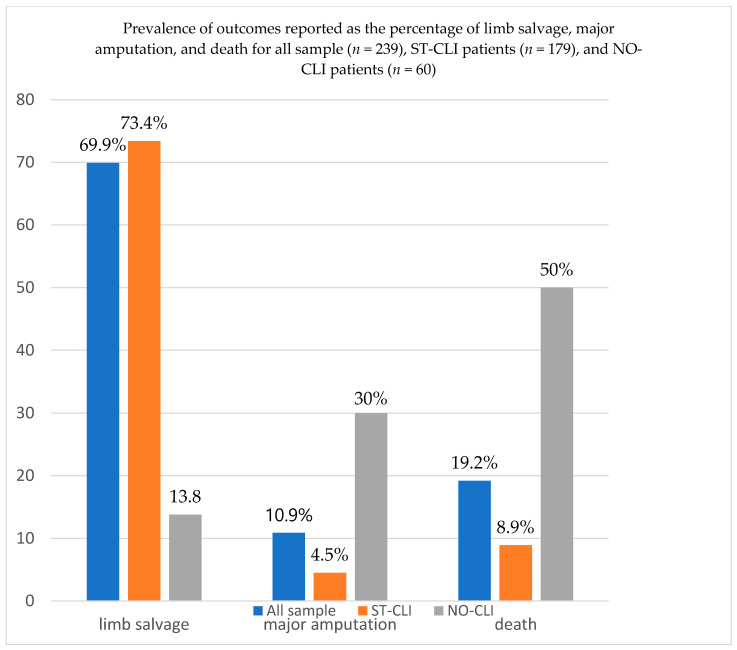
The 1 year outcomes for the whole population, ST-CLI patients, and NO-CLI patients were, respectively, as follows: limb salvage (69.9%, 73.4%, and 13.8%), major amputation (10.9%, 4.5%, and 30%), and death (19.2%, 8.9%, and 50%). ST-CLI: successfully treated critical limb ischemia; NO-CLI: no-option critical limb ischemia.

The 1 year outcomes for the whole population were 167 (69.9%) limb salvages, 26 (10.9%) major amputations, and 46 (19.2%) deaths (see Figure 1).

The 1 year outcomes for ST-CLI and NO-CLI patients were, respectively, as follows: limb salvage (73.4% vs. 13.8%, *p* < 0.0001), amputation (4.5% vs. 30%, *p* = 0.0001), and mortality (8.9% vs. 50%%, *p* < 0.0001) (see Figure 1).

For the multivariate analysis of all predictors found at univariate analysis, no-option CLI (OR 10.3, 95% CI 3.6–17.4; *p* < 0.0001) and dialysis (OR 2.3, 95% CI 1.8–2.9; *p* = 0.02) were independent predictors of major amputation, while no-option CLI (OR 6.1, 95% CI 2.8–9.5; *p* < 0.001), dialysis (OR 5.7, 95% CI 1.9–8.6; *p* = 0.0001), and heart failure (OR 2.5, 95% CI 1.5–4.3; *p* = 0.008) were independent predictors of mortality.

## 4. Discussion

Although many improvements have been achieved in the treatment of diabetic foot patients with PAD [9,10], there are still several cases of persons with NO-CLI. 

In our study, where ischemic DFU patients were managed using a multidisciplinary team approach, including expert interventional radiologists and vascular surgeons, we reported revascularization failure in approximately 25% of patients. The current rate of failed revascularization is apparently higher than literature data. Faglia et al. reported approximately 5% of failed revascularization in a large cohort of ischemic DF patients [5]. Ferraresi et al. reported 7% in patients with infra-popliteal arterial lesions [11], while Uccioli et al. reported approximately 11% of failed cases on the basis of a cohort of patients included between 2002 and 2007 [12].

This evident increase in cases of failed revascularization in recent years could be related to the more aggressive pattern of CLI in our cohort of patients, probably due to diabetes-related characteristics and the increase in concomitant comorbidities, which can negatively impact atherosclerotic disease. 

According to the above reports, there was a longer diabetes duration in our cohort, in comparison to the populations described by Faglia and Ferraresi (approximately 21 vs. 17 and 15 years, respectively) and a higher rate of ischemic heart disease (approximately 66% vs. 56% and 28%), cerebrovascular disease (approximately 16% vs. 14% and 4%), and mainly ESRD in renal replacement therapy (34% vs. 5.7% and 3%) [5,11].

Similarly, there were fewer patients with ischemic heart disease (41% vs. 66%) and dialysis (14% vs. 34%) in the population described by Uccioli et al., in comparison to our data [12]. A recent study by Dalla Paola et al. reported approximately 8% of NO-CLI patients among 1024 subjects, although fewer patients with ischemic heart disease (approximately 55%) and hemodialysis (19%) were observed in comparison to our sample [13].

According to the purpose of our study, the vascular pattern of NO-CLI patients included in our cohort could be defined in more detail; they reported more affected vessels (average 5 vs. 4), as well as a higher involvement of the tibio-peroneal trunk (50 vs. 30%), BTK (anterior tibial artery 93 vs. 83%, posterior tibial artery 93 vs. 74%, peroneal artery 70 vs. 48%), and BTA (overall 73 vs. 39%, pedal artery 57 vs. 24%, plantar arteries 47 vs. 28%), when compared to patients who achieved successful revascularization. Therefore, they were characterized by several arterial lesions and a specific distal (BTK) and ultra-distal (BTA) pattern of PAD. The involvement of these specific districts of the lower-limb vascular tree was significantly higher than that reported by Ferraresi et al. (approximately 28–36% of below-the-knee and 5–9% of below-the-ankle arterial disease) [11], and the number of vessels affected was higher than that found by Uccioli et al. (average 5 vs. 2.6) [12].

The number of arterial lesions [14] is considered a specific parameter of PAD severity, as well as the involvement of BTA arteries, which identifies a complex pattern of PAD in diabetic patients, justifying the high rate of revascularization failure as already described [4,14,15].

In addition, in the group of NO-CLI, more cases of dialysis were reported, in comparison to the ST-CLI group (60 vs. 26%), and dialysis is a well-known independent risk factor for BTA arterial disease [5,16], together with revascularization failure [17]. This finding reinforces the concept that a more aggressive pattern of PAD in diabetic foot patients could be determined not directly by diabetes per se, but by the presence of concomitant cardiovascular risk factors, mainly dialysis, which lead to a widespread distribution of arterial lesions that very often involve the ultra-distal vessels.

In our current study, NO-CLI patients also showed a higher rate of 1 year amputation (30%) and mortality (50%), in contrast with the ST-CLI group. The data we found on amputation and mortality are more or less similar to or slightly higher than similar observations reported by Faglia and Uccioli [5,12]. In a focused study evaluating only the outcome of a specific sample of patients with unreconstructed chronic CLI, Lepantalo et al. reported a 1 year survival rate of 46%, even though approximately only 50% of those were affected by diabetes [18]. In a similar study where outcomes of patients with DFUs who did not receive vascular intervention were analyzed, Elgzyri et al. reported that 17% of those had major amputation and 33% died unhealed [19].

In addition, in our sample of patients, NO-CLI was an independent predictor of major amputation and mortality. These data confirm that revascularization failure per se is a predictor of worse outcomes, as already reported in the abovementioned studies [5,12,18]. Nonetheless, amputation and mortality appeared not only to be related to the severity of PAD, but also to the presence of concomitant comorbidities. In particular, NO-CLI also showed more cases of dialysis as reported above and more cases of heart failure, which are independent risk factors for mortality in patients with ischemic DFUs [5,20,21,22,23,24].

This study was retrospectively analyzed and data reported were based on the results of a single center where patients are managed by a specialized multidisciplinary foot team, including expert interventional radiologists and vascular surgeons. The sample of patients included was not large, especially for NO-CLI patients. A larger sample could be useful to reinforce these data.

## 5. Conclusions

To our knowledge, this is the first study which specifically describes the characteristics of NO-CLI patients, in comparison to those who received successful revascularization.

A specific pattern of PAD in NO-CLI subjects was identified, and the influence of concomitant comorbidities on the severity of arterial lesions was documented. NO-CLI patients showed a more severe pattern of PAD with distal arterial lesions and worse outcomes than ST-CLI.

Currently, patients defined as NO-CLI are presenting as a new challenge for clinicians, vascular surgeons, and interventional cardiologists/radiologists. Notwithstanding the fact that new extreme approaches for managing these patients are currently available for very skilled and expert vascular surgeons or interventional cardiologists/radiologists, a substantial number of patients are not suitable for treatment, and the rate of major amputation is still high. Therefore, we retain that adequately knowing each characteristic of NO-CLI could be vital in order to improve vascular strategies, as well as revascularization techniques and devices. Through the knowledge of specific characteristics of PAD in ischemic DFU subjects, future research is needed to improve revascularization techniques and outcomes. 

## Figures and Tables

**Table 1:** Demographic, clinical, and ulcer characteristics of all samples, successfully treated critical limb ischemia (CLI) patients (ST-CLI), and no-option CLI patients (NO-CLI). LDL: low-density-lipoprotein; CVD: cerebrovascular disease; ESRD: end-stage renal disease; CRP: c-reactive protein. A *p*-value < 0.5 was considered statistically significant.

Variables	All Sample (*n* = 239)	ST-CLI (*n* = 179)	NO-CLI (*n* = 60)	*p*-Values
Age (years)	68.8 ± 9.6	68.7 ± 9.4	69 ± 10.3	0.8
Sex (man)	173 (72.4%)	127 (70.9%)	46 (76.7%)	0.3
Diabetes type (1)	26 (10.9%)	16 (9%)	10 (16.7%)	0.8
Diabetes type (2)	213 (89.1%)	163 (91%)	50 (83.3%)	0.06
Diabetes duration (years)	21.4 ± 12	21.1 ± 11.8	22.1 ± 13	0.5
HbA1c (%) (mmol/mol)	(7.9 ± 4.2) (63 ± 22)	(7.9 ± 4.2) 63 ± 22	(8 ± 4.3) 64 ± 23	0.6
Hypertension	207 (86.6%)	163 (91.1%)	44 (73.3%)	0.001
Dyslipidaemia	161 (67.3%)	113 (63.8%)	42 (75%)	0.1
LDL cholesterol (mg/dL)	66 ± 19	70 ± 16	35 ± 17	0.0004
Ischemic heart disease	158 (66.1%)	110 (62.1%)	48 (80%)	0.008
Heart failure	96 (40.1%)	58 (32.4%)	38 (63.3%)	<0.0001
CVD	38 (15.9%)	24 (13.7%)	14 (23.3%)	0.09
ESRD	82 (34.3%)	46 (25.7%)	36 (60%)	<0.0001
Active smoking habit	23 (9.6%)	17 (9.7%)	6 (10%)	0.9
Anemia	190 (79.5%)	136 (76%)	54 (90%)	0.01
CRP	68 ± 52	66 ± 55	73 ± 44	0.4
**Ulcer characteristics**				
Dimension (>5 cm^2^)	158 (66.1%)	116 (64.8%)	42 (70%)	0.4
Infection	165 (69%)	127 (70.9%)	38 (63.3%)	0.2
Gangrene	169 (70.7%)	121 (67.6%)	48 (80%)	0.06
Osteomyelitis	124 (51.9%)	90 (50.3%)	34 (56.7%)	0.4
Heel location	56 (23.4%)	34 (19%)	22 (36.7%)	0.006

**Table 2:** Distribution of arterial lesions and vascular findings. CFA: common femoral artery; SFA: superficial femoral artery; TPT: tibio-peroneal trunk; ATA: anterior tibial artery; PTA: posterior tibial artery; BTA: below-the-ankle. TcPO_2_: transcutaneous oximetry. A *p*-value < 0.5 was considered statistically significant.

Variable	All Sample (*n* = 239)	ST-CLI (*n* = 179)	NO-CLI (*n* = 60)	*p*-Values
Vessels affected (*n*)	4.3 ± 1.7	4 ± 1.5	5.2 ± 1.6	<0.0001
Iliac arteries (*n*) (%)	6 (2.5%)	4 (2.2%)	2 (3.3%)	0.6
CFA	2 (0.8%)	2 (1.1%)	0 (0%)	0.3
Profunda artery	6 (2.5%)	6 (3.3%)	0 (0%)	0.06
SFA	193 (80.7%)	143 (79.9%)	50 (83.3%)	0.5
Popliteal artery	79 (33%)	57 (31.8%)	22 (36.7%)	0.4
TPT	84 (35.1%)	54 (30.2%)	30 (50%)	0.006
ATA	204 (85.3%)	148 (82.7%)	56 (93.3%)	0.03
Peroneal artery	128 (53.5%)	86 (48%)	42 (70%)	0.002
PTA	188 (78.7%)	132 (73.7%)	56 (93.3%)	0.0005
BTA arteries	114 (47.7%)	70 (39.1%)	44 (73.3%)	<0.0001
Pedal artery	78 (32.6%)	44 (24.6%)	34 (56.7%)	<0.0001
Plantar arteries	78 (32.6%)	50 (27.9%)	28 (46.7%)	0.008
Baseline TcPO_2_ (mmHg)	16 ± 10	18 ± 10	12 ± 9	0.0001
TcPO_2_ at 1 month (mmHg)	43 ± 13	46 ± 12	33 ± 12	<0.0001

## References

[1] Schaper N.C., Van Netten J.J., Apelqvist J., Bus S.A., Hinchliffe R.J., Lipsky B.A. (2020). IWGDF Editorial Board Practical Guidelines on the prevention and management of diabetic foot disease (IWGDF 2019 update). Diabetes Metab. Res. Rev..

[2] Hinchliffe R.J., Andros G., Apelqvist J., Bakker K., Friederichs S., Lammer J., Lepäntalo M., Mills J.L., Reekers J., Shearman C.P. (2012). A systematic review of the effectiveness of revascularization of the ulcerated foot in patients with diabetes and peripheral arterial disease. Diabetes/Metabolism Res. Rev..

[3] Meloni M., Izzo V., Giurato L., Gandini R., Uccioli L. (2019). Below-the-ankle arterial disease severely impairs the outcomes of diabetic patients with ischemic foot ulcers. Diabetes Res. Clin. Pract..

[4] Meloni M., Izzo V., Giurato L., Lázaro-Martínez J.L., Uccioli L. (2020). Prevalence, Clinical Aspects and Outcomes in a Large Cohort of Persons with Diabetic Foot Disease: Comparison between Neuropathic and Ischemic Ulcers. J. Clin. Med..

[5] Faglia E., Clerici G., Clerissi J., Gabrielli L., Losa S., Mantero M., Caminiti M., Curci V., Quarantiello A., Luppattelli T. (2009). Long-Term Prognosis of Diabetic Patients with Critical Limb Ischemia: A population-based cohort study. Diabetes Care.

[6] (2014). Standards of Medical Care in Diabetes. Majmaah J. Health Sci..

[7] Norgren L., Hiatt W.R., Nehler M.R., Harris K.A., Fowkes F.G. (2007). On behalf of the TASC II Working Group. J. Vasc. Surg..

[8] Aiello A., Anichini R., Brocco E., Caravaggi C., Chiavetta A., Cioni R., Da Ros R., De Feo M., Ferraresi R., Florio F. (2014). Treatment of peripheral arterial disease in diabetes: A consensus of the Italian Societies of Diabetes (SID, AMD), Radiology (SIRM) and Vascular Endovascular Surgery (SICVE). Nutr. Metab. Cardiovasc. Dis..

[9] Hinchliffe R.J., Brownrigg J.R.W., Andros G., Apelqvist J., Boyko E.J., Fitridge R., Mills J.L., Reekers J., Shearman C.P., Zierler R.E. (2016). Effectiveness of revascularization of the ulcerated foot in patients with diabetes and peripheral artery disease: A systematic review. Diabetes Metab. Res. Rev..

[10] Uccioli L., Meloni M., Izzo V., Giurato L., Merolla S., Gandini R. (2018). Critical limb ischemia: Current challenges and future prospects. Vasc. Health Risk Manag..

[11] Ferraresi R., Centola M., Ferlini M., Da Ros R., Caravaggi C., Assaloni R., Sganzaroli A., Pomidossi G.A., Bonanomi C., Natalini G. (2009). Long-term Outcomes after Angioplasty of Isolated, Below-the-knee Arteries in Diabetic Patients with Critical Limb Ischaemia. Eur. J. Vasc. Endovasc. Surg..

[12] Uccioli L., Gandini R., Giurato L., Fabiano S., Pampana E., Spallone V., Vainieri E., Simonetti G. (2010). Long-Term Outcomes of Diabetic Patients with Critical Limb Ischemia Followed in a Tertiary Referral Diabetic Foot Clinic. Diabetes Care.

[13] Paola L.D., Cimaglia P., Carone A., Scavone G., Boscarino G., Bernucci D., Sbarzaglia P., Censi S., Ferrari R., Campo G. (2019). Limb salvage in diabetic patients with no-option critical limb ischemia: Outcomes of a specialized center experience. Diabet. Foot Ankle.

[14] Graziani L., Silvestro A., Bertone V., Manara E., Andreini R., Sigala A., Mingardi R., De Giglio R. (2007). Vascular Involvement in Diabetic Subjects with Ischemic Foot Ulcer: A New Morphologic Categorization of Disease Severity. Eur. J. Vasc. Endovasc. Surg..

[15] Ferraresi R., Mauri G., Losurdo F., Troisi N., Brancaccio D., Caravaggi C., Neri L. (2018). BAD transmission and SAD distribution: A new scenario for critical limb ischemia. J. Cardiovasc. Surg..

[16] Hata Y., Iida O., Takahara M., Asai M., Masuda M., Okamoto S., Ishihara T., Nanto K., Kanda T., Tsujimura T. (2020). Infrapopliteal Anatomic Severity and Delayed Wound Healing in Patients with Chronic Limb-Threatening Ischemia in the Era of the Global Limb Anatomic Staging System. J. Endovasc. Ther..

[17] (2020). Peripheral arterial disease in persons with renal-diabetic foot ulcers. J. Wound Care..

[18] Lepäntalo M., Mätzke S. (1996). Outcome of unreconstructed chronic critical leg ischaemia. Eur. J. Vasc. Endovasc. Surg..

[19] Elgzyri T., Larsson J., Thörne J., Eriksson K.-F., Apelqvist J. (2013). Outcome of Ischemic Foot Ulcer in Diabetic Patients Who Had no Invasive Vascular Intervention. Eur. J. Vasc. Endovasc. Surg..

[20] Apelqvist J., Elgzyri T., Larsson J., Löndahl M., Nyberg P., Thörne J. (2011). Factors related to outcome of neuroischemic/ischemic foot ulcer in diabetic patients. J. Vasc. Surg..

[21] Gershater M.A., Löndahl M., Nyberg P., Larsson J., Thörne J., Eneroth M., Apelqvist J. (2008). Complexity of factors related to outcome of neuropathic and neuroischaemic/ischaemic diabetic foot ulcers: A cohort study. Diabetology.

[22] Meloni M., Giurato L., Izzo V., Stefanini M., Pampana E., Gandini R., Uccioli L. (2016). Long term outcomes of diabetic haemodialysis patients with critical limb ischemia and foot ulcer. Diabetes Res. Clin. Pract..

[23] Xu L., Qian H., Gu J., Shi J., Gu X., Tang Z.-Y. (2013). Heart failure in hospitalized patients with diabetic foot ulcers: Clinical characteristics and their relationship with prognosis. J. Diabetes.

[24] Meloni M., Izzo V., Giurato L., Cervelli V., Gandini R., Uccioli L. (2018). Impact of heart failure and dialysis in the prognosis of diabetic patients with ischemic foot ulcers. J. Clin. Transl. Endocrinol..

